# The Influence of Social Networks on Adolescent Overweight and Obesity: A Narrative Review

**DOI:** 10.3390/nu18121930

**Published:** 2026-06-15

**Authors:** Maria de Jesus Xavier Aguirre, Moisés Alberto Calle Aguirre, Flavia Cristina Drumond Andrade, Weber Soares, Eva Débora de Oliveira Andrade, Ana Carolina Costa Campos Mota, Mércia Maria de Santi

**Affiliations:** 1 Graduate Program in Demography, Federal University of Rio Grande do Norte, Natal 59078-900, Brazil; marajxavier@hotmail.com; 2 School of Social Work, University of Illinois at Urbana-Champaign, Urbana, IL 61801, USA; fandrade@illinois.edu; 3 Institute of Geosciences, Federal University of Minas Gerais, Belo Horizonte 31270-901, Brazil; weber.igc@gmail.com; 4 Department of Nutrition, Federal University of Rio Grande do Norte, Natal 59078-900, Brazil; evaandradenutri@outlook.com; 5 Graduate Program in Health Sciences, Federal University of Rio Grande do Norte, Natal 59078-970, Brazil; ana.costa.094@ufrn.edu.br; 6 Health School, Federal University of Rio Grande do Norte, Natal 59078-970, Brazil; mercia.santi@ufrn.br

**Keywords:** adolescents, social networks, obesity, dietary habits, physical activity

## Abstract

This narrative review examines the influence of social networks on dietary habits and physical activity, and their relationship to overweight and obesity in adolescents. The study is based on a comprehensive literature search across PubMed, Scopus, EMBASE, PsycINFO, and Web of Science databases, covering the period 2009–2022, and is complemented by a critical analysis of contemporary evidence. The included studies involved adolescents aged 12 to 19 and assessed associations among social networks, body weight, diet, and physical activity, using statistical methods based on social network analysis (SNA). No language restrictions were applied. The results demonstrated that friendship networks significantly influence adolescents’ body weight, eating behaviors, and physical activity levels. Specifically, boys exhibited similar unhealthy food consumption patterns within their networks, while girls’ networks showed similarities in sedentary activities. This review highlights that adolescents’ social networks play a relevant role in weight-related behaviors, with their influence varying based by gender. These findings underscore the need to consider such gender-specific effects when developing prevention and treatment strategies for obesity in this age group.

## 1. Introduction

Adolescent overweight and obesity stand as one of the most pressing public health challenges of the 21st century. Data from the World Health Organization [1] indicate that in 2022, over 390 million children and adolescents aged 5 to 19 were overweight. The prevalence of overweight and obesity in this age group increased significantly, from 8% in 1990 to 20% in 2022, with similar growth rates in both sexes: 19% of girls and 21% of boys were overweight in 2022. While only 2% of children and adolescents (31 million) were obese in 1990, this figure rose to 8% (160 million) in 2022 [1]. The persistent failure of global efforts to curb obesity highlights its severity as a major public health issue, particularly in adolescence, where it serves as a key risk factor for chronic non-communicable diseases such as cardiovascular diseases, type 2 diabetes, and certain types of cancer later in life [2,3,4].

Obesity is a multifactorial and difficult-to-control disease that demands innovative strategies for its management. While interventions focused on individual choices—such as healthy diets, physical activity, and health-related behaviors—are important, their impact remains limited when implemented in isolation [5,6]. Addressing obesity effectively requires comprehensive strategies and practical actions beyond individual-level interventions. The interventions must be integrated with broader efforts to counteract the obesogenic environment and the diverse sociocultural and structural influences influencing obesity, including socioeconomic, psychological, behavioral, cultural, and environmental factors [6,7,8]. For this reason, the weight of the sociocultural environment, exemplified by exposure to fast-food establishments [9], has been the subject of growing research. Among the biological factors implicated in this multifactorial condition, genetic predisposition, exercise duration and intensity, and dietary composition play important and often underappreciated roles [10]. Moreover, the relationship between excess body weight and diminished general health outcomes—including reduced quality of life, impaired mobility, and increased burden of chronic disease—reinforces the urgency of comprehensive prevention strategies.

Beyond the individual, biological, and contextual dimensions that underpin the complexity of obesity, the social relationship environment also plays a crucial role. Within this environment, ideas and influences about health and obesity-related behaviors circulate [11], shaping an individual’s network of contacts [12]. The current environment is saturated with advertisements [13] and digital marketing [14] for cheap, low-nutrient-quality, and calorie-dense meals, which significantly contribute to an obesogenic environment [15,16]. Additionally, poor habits, such as the immoderate use of electronic devices, are positively associated with overweight and obesity in adolescents [17,18]. In this context, peer/colleague social networks strongly influence students’ behaviors regarding physical activity [19], dietary habits [20], alcohol consumption [21], and academic performance [22]. Peer influence has been shown to be more prominent in children’s healthy eating behaviors than sibling influence, although the latter becomes more relevant in the context of family meals [23].

Social networks—defined in this review as the structured sets of interpersonal ties, including friendship, peer, and colleague relationships, in which an adolescent is embedded—significantly influence weight-related behaviors. The terms “social network,” “friendship network,” and “peer network” are used throughout this article to refer to these interpersonal relationship structures, following the usage of each cited study. In the case of adolescents, networks operate, according to Chung et al. [24], through four main mechanisms: visual appeal, content dissemination, socialized digital connections, and adolescent influencers. Platforms such as Instagram and Facebook have demonstrated the capacity to promote both healthy eating habits and relevant eating behaviors [24]. Evidence shows that social support and social ties are decisive elements in weight loss, and platforms such as Facebook and Twitter are widely used to share information and provide emotional support [25].

Affiliation with peer/family groups significantly shapes adolescents’ dietary and physical activity patterns. Mutual influence within these groups, exerted through social networks, creates a complex web of interactions that can affect young people’s health positively or negatively [11,26,27]. Adolescent friendship groups’ behavioral norms, physical activity practices, and dietary habits can spread through social networks and influence others. This sharing of information, ideas, and behaviors is an important health predictor, affecting, for instance, the likelihood of developing overweight or obesity [28,29].

Structurally, a social network consists of a finite set of actors and their interrelationships [30,31] and can be studied through Social Network Analysis (SNA). This methodological tool enables understanding of health and disease phenomena [21], revealing, for example, how adolescent relationships influence obesity-related behaviors [22]. To better understand the relationship between adolescents’ social networks and overweight/obesity, SNA often employs two methods: Exponential Random Graph Models (ERGMs) and the Stochastic Actor-Based Model (SABM) [32]. Although both investigate how network members interact and the dynamics of influence among them [33], these models are based on distinct statistical assumptions, leading to different types of inference and the ability to address different research questions [34].

ERGMs are frequently used to analyze cross-sectional and longitudinal networks in hypothesis-testing scenarios [35] and, in most cases, focus on small or specific networks [35,36]. On the other hand, SABM [37] is a probabilistic computational simulation model that estimates influences [38] among agents in a system, considering their individual properties and the transmission of behaviors [39]. As a class of statistical models for longitudinal data in social networks [40], SABM assumes that network change is a continuous process observed at discrete moments [41]. In summary, these statistical models for social networks enable the study of complex social phenomena, reveal patterns of relationships among social actors, and deepen the understanding of the interdependence between ties and individuals [28].

This narrative review examines the influence of social networks on adolescents’ health habits (diet and physical activity) and their relationship to overweight and obesity. It aims to fill a gap in research on the interaction of these factors, provide insights for future studies.

## 2. Materials and Methods

The narrative review was conducted using the scientific databases PubMed, Scopus, EMBASE, PsycINFO, and Web of Science databases, covering research published from 2009 to 2022, and was supplemented by a critical analysis of contemporary evidence. The data search was performed using a combination of keywords related to social networks, dietary habits, physical activity, and their relationship with overweight and obesity in adolescents. The included studies assessed the associations between social network weight, diet, and physical activity among adolescents aged 12 to 19 years old across all BMI categories (normal weight, overweight, or obese), utilizing statistical methods based on social network analysis (SNA). Titles and abstracts were initially screened to identify studies that met the inclusion criteria, specifically those evaluating adolescents across all. Studies considered relevant proceeded to full-text reading for final selection. There were no language restrictions.

Two reviewers independently conducted a search for studies published between 2009 and 2022 in the PubMed, Scopus, EMBASE, PsycINFO, and Web of Science databases, with no language restrictions. The search strategies included the keywords “Social Network” or “Social Networks,” “obesity” or “overweight,” and “adolescent” or “youth,” as well as MeSH terms, as detailed in Supplementary Material File S1. Duplicate records were manually excluded.

From the selected studies, the following data were extracted: lead author, year, and language of publication; country of data collection; sample characteristics (age, sex, size, relevant health conditions); type of social network analyzed; number of alter egos (if applicable); methods for assessing network types; network effects on BMI; description of results and relevant conclusions; main findings; study quality assessment method; and reported limitations.

## 3. Results

After removing 76 duplicates, 10,808 articles underwent title and abstract screening, of which 33 were selected for full-text assessment. Following evaluation based on predefined methodological and eligibility criteria, 26 articles were excluded, resulting in seven studies included in the final narrative synthesis. A PRISMA-style flowchart summarizing the study selection process is presented in Supplementary Material Figure S1.

### 3.1. Overview and Characteristics of Included Studies

Table 1 presents the characteristics of the 7 studies included in the review, published in English between 2010 and 2022. Two studies were conducted in Australia, two in European countries (England, Germany, the Netherlands, Sweden, and Spain), one in the United States of America, one in China (Taiwan), and one in South Korea.

The sample sizes ranged from 99 to 18,133 participants, with ages between 12 and 18 and a predominance of female participants. Three studies included exclusively female samples [43,44,45], while the remaining four included participants of both sexes [42,46,47,48].

### 3.2. Relationship Between Social Networks and Weight-Related Behavior

Table 2 summarizes the methods, objectives, main findings, and limitations of the seven studies included in this review [42,43,44,45,46,47,48]. Five studies used the Stochastic Actor-Based Model [43,44,45,47,48] and two adopted the Exponential Random Graph Model [42,46].

Among the studies that employed SABM, two adopted a cross-sectional design [44,47], and three used a longitudinal design [43,45,48]. The study conducted in Australia [44] among female high school students investigated the influence of friendship groups on the development and maintenance of body image and eating disorders. It revealed a tendency for girls to choose friends with similar levels of body dissatisfaction and bulimic behaviors but different dietary habits.

The study conducted in Spain [47] analyzed physical activity, its relationship with overweight and obesity, and the structure of friendship networks among adolescents of both sexes who participated in team sports. No significant relationship between physical activity and weight was observed in the overall sample [47]. However, girls who were overweight were more likely to report high levels of physical activity.

Two longitudinal studies that used SABM investigated physical activity networks [43,44]. The study conducted in the United States [43] employed social network analysis to examine the relationship between BMI, physical activity, and friendship groups. The results indicate that, over time, adolescents maintained BMI and physical activity levels similar to their friends, highlighting the influence of friendship ties on sports practices [43].

The study [45] conducted in South Korea examined the influence of friendship relationships on female adolescents’ dietary and physical activity habits. Girls were more likely to share dietary habits with their friends than to engage in physical activity, indicating a stronger peer influence on the dietary choices of Korean adolescents, particularly in the context of shared meals.

Another longitudinal study conducted in Taiwan [48] used the SABM with adolescents of both sexes to assess the influence of peers’ lifestyles on body weight. The results suggest a strong tendency toward homophily, as adolescents choose friends with similar weight and lifestyle characteristics. However, no evidence of peer influence on weight change was found.

As for the two studies that employed ERGM, one adopted a cross-sectional design [42], and the other a longitudinal design [46]. The cross-sectional study with Australian adolescents investigated the relationship between obesity-related behaviors and friendship networks. The results suggest that female friends exhibit similar behaviors related to electronic device use. In contrast, male friends tend to have similar dietary habits, particularly regarding consuming calorie-dense foods. Popularity (measured by the receipt of ties) is also associated with certain behaviors, with gender-specific effects and variations across networks, indicating the influence of social dynamics on obesity among adolescents [42].

Conducted in four European countries (England, Germany, the Netherlands, and Sweden), the longitudinal study [46] that used ERGM investigated the association between adolescents’ BMI and that of their friends. In addition to indicating that BMI clustering is more pronounced in strong friendships and among same-sex adolescents [46], the study suggests that BMI similarity influences friend selection.

### 3.3. Association Between Social Networks and Overweight/Obesity

The association between overweight/obesity and social networks was observed in three studies [42,43,44]. Two of these studies [42,43] demonstrated that adolescents’ weight, including their BMI and physical activity levels, is influenced by friendship ties. Specifically, evidence suggests a behavioral contagion effect, as study [42] showed that friends tend to share sedentary habits related to screen use, including video/computer and internet activities. In contrast, Korean adolescents [45] were less likely to share physical activities with their friends, highlighting how cultural contexts may modulate network influences.

Four studies [44,46,47,48] investigated school friendship networks but found no association between these networks and overweight/obesity. This divergence in the literature underscores the complexity of social influence, suggesting that network effects may be task-specific or mediated by variables not captured in all designs.

Marqués-Sánchez et al. [47] found a significant association between participation in team sports and the intensity of social contacts (centrality). Regarding centrality, a gender -based stratification emerged: women participating in team sports tended to have lower centrality values, while men had higher centrality values.

Two studies [44,48] highlighted the importance of friendship selection during adolescence, emphasizing the role of homophily in body image and eating disorders. The findings suggest that friends are chosen based on similarities in weight, lifestyle, body dissatisfaction, and bulimic behaviors [48]. This mechanism of selection indicates that social clusters may reinforce pre-existing behaviors rather than solely inducing new ones.

Furthermore, Grund and Tatum explored gender differences in body image, eating habits, and physical activity norms [46], revealing a significant BMI clustering among close friends and same-sex adolescents. Supporting the idea that these patterns extend into later developmental stages, Luo et al. [49] identified significant gender-based homophily in unhealthy weight control behaviors among first-year university students. Their study [49] indicated that these clusters are associated with psychological factors, such as an incorrect self-perception of obesity, highlighting the importance of gender homophily in the structural organization of these health behaviors.

## 4. Discussion

This review analyzed seven studies [42,43,44,45,46,47,48] on how adolescent friendship networks relate to diet, physical activity, and obesity. In four studies, no association was found between school-based social networks and overweight/obesity.

The Spanish study [47] found that participation in team sports was linked to degree centrality. Among girls, greater contact and sports involvement were tied to lower centrality, while boys with fewer contacts showed higher centrality.

These findings suggest gender moderates the link between social networks and behavior, highlighting the need for more research. Degree centrality helps identify influential adolescents who can spread habits like diet and exercise, influencing obesity within networks.

Savona et al. [50] identified three feedback loops influencing adolescent obesity: (1) commercial promotion of unhealthy diets, (2) links between mental health and food intake, and (3) social media’s impact on body image and exercise. Exposure to food ads increased consumption, while stress and influencer-driven image pressure reduced motivation to exercise.

The Taiwan study [48] found no peer effect on weight change but did show that adolescents select friends with similar weight and lifestyles. In Australia [49], girls chose friends with similar body dissatisfaction and bulimic traits. These results underscore the role of friendship selection in body image issues.

Studies from Taiwan [48] and Australia [44] show that adolescents choose friends with similar weight and behaviors, highlighting how social validation shapes health choices. This pattern, known as BMI homophily, is theoretically grounded in the social selection process, whereby individuals preferentially form ties with others who share similar phenotypic and behavioral characteristics [46]. Socioeconomic factors may amplify this dynamic: shared family income levels can align dietary environments and lifestyle routines, thereby increasing the likelihood of friendship formation among adolescents with similar weight profiles [10]. Regarding the apparently counterintuitive finding that overweight girls reported higher levels of physical activity [47], this may reflect compensatory behavior—a response to social stigma or medical guidance—rather than indicating that higher activity uniformly produces lower BMI in this population.

The study in four European countries [46] found stronger BMI clustering among close, same-sex friends. Though no direct link to obesity was found, gender differences in body image and health norms emerged. Three other studies [42,43,44] did find associations with social networks.

Social influence in networks occurs through interaction, where beliefs and behaviors shift in response to others [51,52]. In adolescent networks, diet, exercise, and other habits are shared through these dynamics, shaping health-related behaviors.

De la Haye et al. [42] found that male students in Australia shared similar patterns of unhealthy eating, with popularity linked to the consumption of snacks and fast food. Popular boys influenced peers’ habits. Exposure to social media also increased the intake of unhealthy foods and decreased fruit and vegetable consumption by triggering reward-related brain activity [53,54].

Unlike other studies, Chung et al. [45] found that Korean adolescents’ diets were linked to their friends’ characteristics, such as normal weight and healthy habits. This suggests overweight teens may adopt better behaviors through positive peer influence. The mechanisms through which social networks shape obesity-related behaviors operate along several pathways. Normative influence leads peers to align their eating and activity patterns with those perceived as desirable within the group. Behavioral modeling enables direct imitation of friends’ dietary choices—particularly visible in shared meals, as documented among Korean adolescents [45]. Social reinforcement, both positive (peer approval) and negative (stigma), can either encourage or discourage weight-management behaviors. Understanding which mechanisms predominate in specific cultural and socioeconomic contexts is essential for designing targeted, effective interventions.

In Australia [42], planned sports helped form friendships across genders. In contrast, Korean adolescents [45] mainly shared physical education classes with friends during school hours.

A study in Northern Ireland [55] found that peer influence on physical activity varies by gender: boys are influenced by their friend groups, while girls are more affected by their best friends. This may reflect gender norms, where boys form broader networks shaped by group pressure, and girls prioritize intimate, reciprocal bonds, leading to stronger influence from close friends.

De la Haye et al. [42] found that adolescents shared similar screen-related sedentary habits, like gaming and browsing, indicating peer influence. Korean teens also showed sedentary behavior, likely due to academic demands, but without a clear link to obesity [45].

Simpkins et al. [43] showed that friendship networks consistently influence adolescents’ physical activity over time, even when controlling for BMI, reinforcing the idea that social ties shape exercise habits during adolescence.

Furthermore, Fujimoto et al. [56] found that friendships in Southern California influenced adolescents’ sports participation more than shared sports interests, suggesting that social networks played a dual role, both encouraging and discouraging activity through group connections. These findings align with recent evidence highlighting that social network dynamics and gender are significant factors in adolescent behavior, where homophily often contributes to the selection and maintenance of weight-related habits [49,57].

Three studies conducted in Australia [42] the United States [43], and South Korea [45], respectively, demonstrated the influence of friendship relationships on adolescent behavior and identified similarities between adolescents of both sexes regarding leisure activities and dietary habits [42]. This influence, however, transcends purely behavioral aspects, as evidenced by Bozzola et al. [58] who mapped a broader panorama of risks associated with social networks in adolescence, including depression (27.9%), eating disorders (22.1%), cyberbullying (22.1%), psychological issues (20.6%), sleep disturbances (19.1%), addiction (14.7%), anxiety (14.7%), and sexual problems (13.2%) [58]. The American study [43] highlighted the impact of school friendships on both BMI and physical activity levels among youth. In the South Korean school context [45], the practice sharing of meals underscored the mutual influence among adolescents. Observing these interactions during lunch highlights how dietary habits are shaped within the school environment and reinforces the idea that each individual is affected by the dynamics and influences of their social circle.

Three of the seven studies analyzed in this review demonstrated associations between social networks and overweight/obesity. According to these studies, social networks play a crucial role in disseminating behaviors and forming norms and values. Individual health is thus directly affected by relational dynamics, as health-related behaviors, such as dietary patterns and physical activity habits, are shaped by interactions and norms established within social networks [59,60,61]. In this context, Aguirre et al. [62] observed that in specific environments such as rural areas, these dynamics are linked to family influences, where high social centrality within the network may be associated with a higher likelihood of overweight. In other words, these behaviors serve as mechanisms of social influence within the network, resulting in direct consequences for individual health outcomes [61].

Although the other four studies did not identify direct associations between social networks and overweight/obesity, it is important to highlight some relevant findings they brought to light:

Generalized homophily shapes friendships, as adolescents often bond with peers of similar body weight and lifestyles [48]. This is especially evident among girls, who form ties based on shared body dissatisfaction and bulimic traits, even when their diets differ [44]. Recent research expands this perspective by showing that peer effects are gender-dependent; specifically, male students appear particularly responsive to the weight of their female friends. These findings suggest that gender-targeted interventions, particularly those focused on females, may be more effective in reducing overall school BMI than gender-neutral approaches [63]. Grund and Tatum [46] found that adolescents in four European countries tend to befriend peers with similar BMI. Their comparative approach highlights how cultural factors shape the link between social networks and weight-related behaviors. Marqués-Sánchez et al. [47] found that team sports participation affects network position, with girls tending toward peripheral roles and boys occupying central positions, showing gender differences.

In this context of social influences on weight-related behaviors, Castro and Osório [64] provide a crucial additional perspective by analyzing blogs of Portuguese and Brazilian adolescents. Their findings reveal how social pressure and family conflicts catalyze the internalization of thinness ideals, manifesting in disruptive behaviors such as extreme diets and self-harm. The authors identified that these virtual spaces function as environments of mutual validation, where adolescents share content ranging from weight-loss tips to “thinspiration,” highlighting how exposure to pro-anorexia content can amplify pre-existing vulnerabilities and underscoring the importance of interventions that consider the impact of digital media on adolescent health [64].

### Strengths and Limitations

This narrative review possesses distinct strengths. By adopting a narrative synthesis approach, this study provides a comprehensive qualitative synthesis of social complexities that strictly numerical meta-analyses might not fully capture.

However this review has limitations. First, three studies [42,43,45] relied on small, school-based networks (3–10 friends), excluding potentially influential relationships with parents, teachers, and peers outside the school context. Second, reliance on self-reported weight and height in some studies [43,48] may have introduced measurement bias, as participants may have over-reported healthier values. Third, the temporal scope of the evidence is restricted to studies published up to 2022.

Given that the manuscript was submitted in 2026, a four-year gap exists that may not capture recent developments in adolescent social network dynamics—particularly the accelerated transformation of digital environments following the COVID-19 pandemic. This gap reflects the original search protocol and the decision to ensure search replicability rather than a substantive omission. Nonetheless, the theoretical and methodological principles underlying this review remain applicable, and the gap itself signals a productive avenue for future research. Fourth, the small number of included studies (*n* = 7) from the screening process) reflects stringent eligibility criteria intended to ensure methodological comparability; however, it may constrain the generalizability of the conclusions.

Heterogeneity in study designs, populations, and outcome measures further limits cross-study inference. Fifth, the geographic concentration of included studies in high-income countries (Australia, the USA, European nations, Taiwan, and South Korea) limits the applicability of the findings to low- and middle-income settings, where food environments, cultural norms around body weight, and adolescent social dynamics differ considerably. Sixth, none of the included studies systematically accounted for race/ethnicity or family income as potential confounders or moderators of the social network–obesity relationship. These structural factors may independently shape both friendship formation—through residential or school segregation—and dietary patterns—through differential access to healthy foods—and their omission represents an important limitation for future investigation.

Despite these limitations, this review contributes meaningful evidence on how social networks shape obesity-related behaviors in adolescents. The findings point to the value of targeting peer dynamics in prevention strategies: rather than focusing exclusively on individual behavior change, interventions should consider the network context in which behaviors are formed and reinforced. Friendship networks may serve as vehicles for health promotion by identifying and engaging socially central adolescents with a high degree of centrality as peer behavioral models. School-based programs that incorporate peer-led components, shared physical activity, and group dietary practices offer promising frameworks for reshaping network norms. At the same time, intervention design must guard against the unintended reinforcement of unhealthy behavioral norms through the same network dynamics that interventions seek to harness.

Schools are key spaces for applying SNA to study how adolescents adopt dietary and physical activity habits [62,65]. Gender differences are evident, with more eating disorders in girls [66]. Health impacts vary with obesity severity, including differences in cholesterol, HDL, and triglycerides [67]. Mapping peer networks helps reveal how relationships shape behavior, aiding the design of socially grounded health interventions.

Health professionals, especially nutritionists, should consider adolescents’ social environments when addressing obesity. As a chronic condition, it requires personalized, integrated care. Future studies with larger, diverse samples are needed to better understand social network influences.

In conclusion, addressing adolescent obesity requires health professionals to consider social networks. Effective interventions must be integrated and tailored. Future research with broader, representative samples is crucial to deepen understanding and improve outcomes.

## 5. Conclusions

This narrative review provides an integrative understanding of how social networks shape weight-related behaviors in adolescence. Evidence from seven empirical studies—employing SABM and ERGM as principal analytical frameworks—does not support a consistent or causal association between social networks and overweight/obesity outcomes. Rather, it suggests that network processes operate primarily through behavioral pathways, particularly dietary patterns and physical activity, functioning as intermediate systems that influence behavioral risk rather than acting as direct determinants of weight status. The synthesis of findings reflects methodological heterogeneity, reliance on school-bounded samples, and the specific number of eligible studies.

Two mechanisms are central to understanding how these processes unfold. The first is homophily, or social selection, whereby adolescents preferentially form ties with peers who share similar weight profiles and lifestyle characteristics. The second is social influence, through which dietary habits, physical activity patterns, and sedentary behaviors are reinforced and diffused through friendship ties over time. These processes are not mutually exclusive, and disentangling their relative contributions constitutes a central methodological challenge for future research. Gender further moderates both mechanisms: boys tend to exhibit network-level similarities in high-calorie food consumption, whereas girls show stronger clustering around sedentary behaviors, body dissatisfaction, and disordered eating patterns. The absence of consistent associations with BMI reflects the complexity of obesity as a multifactorial condition in which social networks represent one layer within a broader causal structure.

From a public health perspective, these findings highlight the potential of network-informed prevention strategies. Rather than relying exclusively on individual behavior change, interventions should target the relational contexts in which adolescent health behaviors are formed and reinforced. School settings offer privileged opportunities for such approaches, as they concentrate friendship networks and allow for the systematic identification of high-centrality adolescents who may serve as behavioral models for their peers. Such approaches must, however, be carefully designed to avoid inadvertently reinforcing the unhealthy behavioral norms they seek to disrupt.

Future research should prioritize longitudinal designs with representative and diverse samples that extend beyond school boundaries to capture the full range of adolescent relational contexts, including digital social networks, family ties, and community-level interactions. Advanced network models—particularly those integrating ERGMs and SABM with contextual and socioeconomic covariates—will be essential for unpacking causal mechanisms. Special attention should be given to understudied populations in low- and middle-income settings, where food environments, cultural body norms, and network structural properties may differ considerably from the predominantly high-income samples currently represented in the literature. In summary, adolescent obesity cannot be fully understood without considering the relational environments in which behaviors are formed. Integrating social network perspectives into both research design and intervention frameworks represents a critical step toward more effective and contextually grounded public health strategies.

## Figures and Tables

**Table 1:** Characteristics of the studies included in the narrative review.

Authors (Year)	Study Design	Country	Sample Size	Studied Genders
K. de la Haye et al. (2010) [42]	Cross-sectional	Australia	385	Male and female
Simpkins, Schaefer, Price, and Vest (2013) [43]	Longitudinal	USA	1896	Female
Rayner, Schniering, Rapee, Taylor, and Hutchinson (2013) [44]	Cross-sectional	Australia	1197	Female
Chung et al. (2019) [45]	Longitudinal	Korea	99	Female
Grund and Tatum (2019) [46]	Longitudinal	England, Germany, the Netherlands, and Sweden	18,133	Male and female
Marqués-Sánchez et al. (2021) [47]	Cross-sectional	Spain	235	Male and female
Lee et al. (2022) [48]	Longitudinal	Taiwan	2409	Male and female

**Table 2:** Outcome measures, social network characteristics and statistical models of the studies included in the review.

Authors (Year)	Ego	Alter	Types of SNA	Main Results	Limitations
K. de la Haye et al. (2010) [42]	Adolescent	Friends	Exponential Random Graph Models (ERGM	There were evidence male friends tended to be similar in their consumption of high-calorie foods, and adolescent school friends were found to be similar in some obesity-related behaviors, particularly leisure activities	This study does not include friends outside of school and family.
Simpkins, Schaefer, Price, and Vest. (2013) [43]	Adolescent	Friends	Stochastic actor-based model (SABM)	There was evidence that friends influence BMI and physical activity. Friendships were more likely among adolescents who engaged in greater physical activity and were similar to one another in BMI and physical activity.	Analyses were limited to five female and five male friends. It did not consider friendships outside of school as family and other friendships. Most measures were based on adolescents’ self-reported weight and height.
Rayner, Schniering, Rapee, Taylor, and Hutchinson (2013) [44]	Adolescent	Friends	Stochastic actor-based model (SABM)	Evidence indicates that friends influence BMI and physical activity. Friendships were more likely among adolescents who engaged in greater physical activity and were similar in BMI and physical activity.	This study focused exclusively on adolescent girls and did not consider male friendships, family members, or friendships outside of school.
Chung et al. (2019) [45]	Adolescent	Friends	Stochastic actor-based model (SABM	Adolescents’ dietary behaviors were connected with their classroom-based peer networks; however, similar outcomes were not identified for physical activity.	The use of self-report questionnaires can lead to desirability bias, overreporting of healthier behaviors, and underreporting of unhealthy behaviors. Given the reliance on self-reported weight and height based on recall of the annual health assessment, it is possible that participants were biased in their recall of these measurements.
Grund and Tatum (2019) [46]	Adolescent	Friends	Exponential Random Graph Models (ERGM)	Strong evidence for BMI clustering in England, Germany, the Netherlands, and Sweden demonstrated that adolescents tend to be friends with people of similar BMI.	This study does not consider different types of relationships with parents, teachers, or other adolescents outside of the classroom.
Marqués-Sanchez et al. (2021) [47]	Adolescent	Friends	Stochastic actor-based model (SABM)	There was no significant relationship between physical activity and weight status in the total sample, but among female participants, those with overweight status had higher odds of reporting high levels of physical exercise.	The use of self-report questionnaires can lead to desirability bias, overreporting of healthier behaviors, and underreporting of unhealthy behaviors. Given the reliance on self-reported weight and height based on recall of the annual health assessment, it is possible that participants were biased in their recall of these measurements.
Lee et al. (2022) [48]	Adolescent	Friends	Stochastic actor-based model (SABM)	Adolescents with similar weight status, athletic performance, internet use, and social jetlag were likely to befriend each other. There was no evidence supporting friends’ influence on an adolescent’s weight. Did not observe any peer influence on changes in adolescents’ weight status.	This study only refers to up to three friends and disregards any colleagues outside of school.

## Data Availability

No new data were created or analyzed in this study. Data sharing is not applicable to this article.

## Supplementary Materials

The following supporting information can be downloaded at: https://www.mdpi.com/article/10.3390/nu18121930/s1, Figure S1: PRISMA-Style flow diagram of study selection. File S1: Full electronic search strategy for PubMed, Scopus, Physiinfo, Web of science and EMBASE databases.

## Abbreviations

The following abbreviations are used in this manuscript:

ERGM	Exponential Random Graph Models
SABM	Stochastic Actor-Based Model
SNA	Social Network Analysis
NOS	Newcastle-Ottawa Scale
AHRQ	Healthcare Research and Quality
GRADE	Grading of Recommendations Assessment, Development, and Evaluation

## References

[1] World Health Organization (2023). Obesity and Overweight.

[2] Lauby-Secretan B., Scoccianti C., Loomis D., Grosse Y., Bianchini F., Straif K. (2016). Body fatness and cancer viewpoint of the IARC Working Group. N. Engl. J. Med..

[3] Llewellyn A., Simmonds M., Owen C.G., Woolacott N. (2016). Childhood obesity as a predictor of morbidity in adulthood: A systematic review and meta-analysis. Obes. Rev..

[4] Ng M., Fleming T., Robinson M., Thomson B., Graetz N., Margono C., Mullany E.C., Biryukov S., Abbafati C., Abera S.F. (2014). Global, regional, and national prevalence of overweight and obesity in children and adults during 1980–2013: A systematic analysis for the Global Burden of Disease Study 2013. Lancet.

[5] Wharton S., Lau D.C., Vallis M., Sharma A.M., Biertho L., Campbell-Scherer D., Adamo K., Alberga A., Bell R., Boulé N. (2020). Obesity in adults: A clinical practice guideline. Can. Med. Assoc. J..

[6] Brown T., Moore T.H., Hooper L., Gao Y., Zayegh A., Ijaz S., Elwenspoek M., Foxen S.C., Magee L., O’Malley C. (2019). Interventions for preventing obesity in children. Cochrane Database Syst. Rev..

[7] Lemstra M., Bird Y., Nwankwo C., Rogers M., Moraros J. (2016). Weight loss intervention adherence and factors promoting adherence: A meta-analysis. Patient Prefer. Adherence.

[8] Hemmingsson E., Nowicka P., Ulijaszek S., Sørensen T.I.A. (2023). The social origins of obesity within and across generations. Obes. Rev..

[9] Van Erpecum C.L., Van Zon S.K.R., Xie T., Snieder H., Bültmann U., Smidt N. (2023). Fast-food environments and BMI changes in the Dutch adult general population: The Lifelines cohort. Obesity.

[10] Kelly A.S., Armstrong S.C., Michalsky M.P., Fox C.K. (2024). Obesity in adolescents: A review. JAMA.

[11] Christakis N.A., Fowler J.H. (2000). The spread of obesity in a large social network over 32 years. N. Engl. J. Med..

[12] Serrano Fuentes N., Rogers A., Portillo M.C. (2019). Social network influences and the adoption of obesity-related behaviours in adults: A critical interpretative synthesis review. BMC Public Health.

[13] Kelly B., Vandevijvere S., Ng S., Adams J., Allemandi L., Bahena-Bahena-Espina L., Barquera S., Boyland E., Calleja P., Carmona-Garcés I.C. (2019). Global benchmarking of children’s exposure to television advertising of unhealthy foods and beverages across 22 countries. Obes. Rev..

[14] Potvin-Kent M., Pauzé E., Roy E.A., Billy N., Czoli C. (2019). Children and adolescents’ exposure to food and beverage marketing in social media apps. Pediatr. Obes..

[15] Popkin B.M., Barquera S., Corvalan C., Hofman K.J., Monteiro C., Ng S.W., Swart E.C., Taillie L.S. (2021). Towards unified and impactful policies to reduce ultra-processed food consumption and promote healthier eating. Lancet Diabetes Endocrinol..

[16] Vercammen K.A., Frelier J.M., Moran A.J., Dunn C.G., Musicus A.A., Wolfson J.A., Bleich S.N. (2019). Calorie and nutrient profile of combination meals at U.S. fast food and fast casual restaurants. Am. J. Prev. Med..

[17] Bakour C., Mansuri F., Johns-Rejano C., Crozier M., Wilson R., Sappenfield W. (2022). Association between screen time and obesity in US adolescents: A cross-sectional analysis using National Survey of Children’s Health 2016–2017. PLoS ONE.

[18] Throuvala M.A., Griffiths M.D., Rennoldson M., Kuss D.J. (2021). The role of recreational online activities in school-based screen time sedentary behaviour interventions for adolescents: A systematic and critical literature review. Int. J. Ment. Health Addict..

[19] Di Guiseppi G.T., Meisel M.K., Balestrieri S.G., Ott M.Q., Clark M.A., Barnett N.P. (2018). Relationships between social network characteristics, alcohol use, and alcohol-related consequences in a large network of first-year college students: How do peer drinking norms fit in?. Psychol. Addict Behav..

[20] Vignery K. (2022). From networked students centrality to student networks density: What really matters for student performance?. Soc. Netw..

[21] Mackey E.R., La Greca A.M. (2007). Adolescents’ eating, exercise, and weight control behaviors: Does peer crowd affiliation play a role?. J. Pediatr. Psychol..

[22] Cruwys T., Bevelander K.E., Hermans R.C. (2015). Social modeling of eating: A review of when and why social influence affects food intake and choice. Appetite.

[23] Ragelienė T., Grønhøj A. (2021). The role of peers, siblings and social media for children’s healthy eating socialization: A mixed methods study. Food Qual. Prefer..

[24] Chung A., Vieira D., Donley T., Tan N., Jean-Louis G., Gouley K.K., Seixas A. (2021). Adolescent peer influence on eating behaviors via social media: Scoping review. J. Med. Internet Res..

[25] Li C., Ademiluyi A., Ge Y., Park A. (2022). Using social media to understand web-based social factors concerning obesity: Systematic review. JMIR Public. Health Surveill..

[26] Du T., Li Y. (2022). Effects of social networks in promoting young adults’ physical activity among different sociodemographic groups. Behav. Sci..

[27] Harmon B.E., Forthofer M., Bantum E.O., Nigg C.R. (2016). Perceived influence and college students’ diet and physical activity behaviors: An examination of ego-centric social networks. BMC Public Health.

[28] Pachucki M.A., Jacques P.F., Christakis N.A. (2011). Social network concordance in food choice among spouses, friends, and siblings. Am. J. Public Health.

[29] Ragelienė T., Grønhøj A. (2020). The influence of peers’ and siblings’ on children’s and adolescents’ healthy eating behavior: A systematic literature review. Appetite.

[30] Amati V., Meggiolaro S., Rivellini G., Zaccarin S. (2018). Social relations and life satisfaction: The role of friends. Genus.

[31] Vörös A., Boda Z., Elmer T., Hoffman M., Mepham K., Raabe I.J., Stadtfeld C. (2021). The Swiss StudentLife Study: Investigating the emergence of an undergraduate community through dynamic, multidimensional social network data. Soc. Netw..

[32] Agneessens F., Trincado-Munoz F.J., Koskinen J. (2022). Network formation in organizational settings: Exploring the importance of local social processes and team-level contextual variables in small groups using Bayesian hierarchical ERGMs. Soc. Netw..

[33] George G., Vega Yon A., Slaughter A., de la Haye K. (2021). Exponential random graph models for little networks. Soc. Netw..

[34] Block P., Koskinen J., Hollway J., Steglich C., Stadtfeld C. (2018). Change we can believe in: Comparing longitudinal network models on consistency, interpretability and predictive power. Soc. Netw..

[35] Huang W., Lusher D., Koskinen J., Robins G. (2012). Exponential Random Graph Models for Social Networks: Theory, Methods, and Applications.

[36] Tolochko P., Boomgaarden H.G. (2024). Same but different. Soc. Netw..

[37] Hipp J.R., Wang C., Butts C.T., Jose R., Lakon C.M. (2015). Research note: The consequences of different methods for handling missing network data in stochastic actor-based models. Soc. Netw..

[38] Snijders T.A.B., Van de Bunt G.G., Steglich C.E.G. (2010). Introduction to stochastic actor-based models for network dynamics. Soc. Netw..

[39] Luke D.A., Stamatakis K.A. (2012). Systems science methods in public health: Dynamics, networks, and agents. Annu. Rev. Public Health.

[40] Diviák T., van Nassau C.S., Dijkstra J.K., Snijders T.A.B. (2022). Dynamics and disruption: Structural and individual changes in two Dutch Jihadi networks after police interventions. Soc. Netw..

[41] Tasselli S., Neray B., Lomi A. (2023). A network centrality bias: Central individuals in workplace networks have more supportive coworkers. Soc. Netw..

[42] Haye K., Robins G., Mohr P., Carlene W. (2010). Obesity-related behaviors in adolescent friendship networks. Soc. Netw..

[43] Simpkins S.D., Schaefer D.R., Price C.D., Vest A.E. (2013). Adolescent friendships, BMI, and physical activity: Untangling selection and influence through longitudinal social network analysis. J. Res. Adolesc..

[44] Rayner K.E., Schniering C.A., Rapee R.M., Taylor A., Hutchinson D.M. (2013). Adolescent girls’ friendship networks, body dissatisfaction, and disordered eating: Examining selection and socialization processes. J. Abnorm. Psychol..

[45] Chung S.J., Ersig A.L., McCarthy A.M. (2019). Diet and physical activity of Korean female adolescents in their peer networks. J. Nurs. Sch..

[46] Grund T.U., Tatum T. (2019). Some friends matter more than others: BMI clustering among adolescents in four European countries. Netw. Sci..

[47] Marqués-Sánchez P., Benítez-Andrades J.A., Calvo Sánchez M.D., Arias N. (2021). The socialisation of the adolescent who carries out team sports: A transversal study of centrality with a social network analysis. BMJ Open.

[48] Lee C.T., Chen T.W., Ubeda Herrera J.J., Yu Y.F., Strong C., Lin C.Y., Chang Y.H., Hsieh Y.P., Lin Y.C., Tsai M.C. (2022). Dynamic weight status changes and peer lifestyles in early adolescence: A social network analysis on a longitudinal cohort of Taiwanese youth. Obes. Res. Clin. Pract..

[49] Luo R., Zhang J., Liu J., Yan H., Chen M., Shang M., Kong L., Tang Y., Hao C., Li J. (2025). Homophily and gender differences in unhealthy weight control behaviors among adolescents: A longitudinal school-based friendship network study. BMC Public Health.

[50] Savona N., Macauley T., Aguiar A., Banik A., Boberska M., Brock J., Brown A., Hayward J., Holbæk H., Rito A.I. (2021). Identifying the views of adolescents in five European countries on the drivers of obesity using group model building. Eur. J. Public Health.

[51] Friedkin N.E., Wasserman S. (2000). A structural theory of social influence. Am. J. Sociol..

[52] Valente T.W., Rogers E.M. (1995). The origins and development of the diffusion of innovations paradigm as an example of scientific growth. Sci. Commun..

[53] Sina E., Boakye D., Christianson L., Ahrens W., Hebestreit A. (2022). Social media and children’s and adolescents’ diets: A systematic review of the underlying social and physiological mechanisms. Adv. Nutr..

[54] Qutteina Y., Hallez L., Raedschelders M., De Backer C., Smits T. (2022). Food for teens: How social media is associated with adolescent eating outcomes. Public Health Nutr..

[55] Montgomery S.C., Donnelly M., Badham J., Kee F., Dunne L., Hunter R.F. (2021). A multi-method exploration into the social networks of young teenagers and their physical activity behavior. BMC Public Health.

[56] Fujimoto K., Snijders T., Valente T. (2018). Multivariate dynamics of one-mode and two-mode networks: Explaining similarity in sports participation among friends. Netw. Sci..

[57] Marqués-Sánchez P., Martínez-Fernández M.C., Benítez-Andrades J.A., Quiroga-Sánchez E., García-Ordás M.T., Arias-Ramos N. (2023). Adolescent relational behaviour and the obesity pandemic: A descriptive study applying social network analysis and machine learning techniques. PLoS ONE.

[58] Bozzola E., Spina G., Agostiniani R., Barni S., Russo R., Scarpato E., Di Mauro A., Di Stefano A.V., Caruso C., Corsello G. (2022). The use of social media in children and adolescents: Scoping review on the potential risks. Int. J. Environ. Res. Public Health.

[59] Berkman L.F., Glass T., Brissette I., Seeman T.E. (2000). From social integration to health: Durkheim in the new millennium. Soc. Sci. Med..

[60] Lin S., Faust L., Robles-Granda P., Kajdanowicz T., Chawla N.V. (2019). Social network structure is predictive of health and wellness. PLoS ONE.

[61] Montgomery M.R., Casterline J.B. (1996). Social learning, social influence, and new models of fertility. Popul. Dev. Rev..

[62] Aguirre M.J.X., Drumond Andrade F.C., Aguirre M.A.C., Justino J.R., Maciel B.L.L. (2023). Social Network, Food Patterns, Physical Activity and Associations with Overweight and Obesity in Adolescents from a School in Rural Brazil. Nutrients.

[63] Comola M., Dieye R., Fortin B. (2025). Heterogeneous peer effects and gender-based interventions for teenage obesity. J. Health Econ..

[64] Sofia Castro T., José Osório A. (2013). “I love my bones!”1–Self-harm and dangerous eating youth behaviours in Portuguese written blogs. Young Consum. Insight Ideas Responsible Mark..

[65] Lima C.V.P., Ywgne J., Thuany M., Araujo R.H.O., Silva E.C.M., Melo J.C.N., Bandeira P.F.R., Luz L.G.O. (2023). What are the correlates of intention to be physically active in Brazilian adolescents? A network analysis. BMC Public Health.

[66] Dias R.G., Rech R.R., Halpern R. (2023). Prevalence and associated factors of eating disorder symptoms in adolescents: A cross-sectional school-based study. BMC Psychiatry.

[67] Bays H.E., Kirkpatrick C., Maki K.C., Toth P.P., Morgan R.T., Tondt J., Christensen S.M., Dixon D., Jacobson T.A. (2024). Obesity, dyslipidemia, and cardiovascular disease: A joint expert review from the Obesity Medicine Association and the National Lipid Association 2024. J. Clin. Lipidol..

