# Supplementary material for: The Influence of Social Networks on Adolescent Overweight and Obesity: A Narrative Review
Source: Nutrients. 2026 Jun 15;18(12):1930. doi: 10.3390/nu18121930 (PMC13305445; doi:10.3390/nu18121930)
Supplement: Supplementary file 1 [file nutrients-18-01930-s001.zip › nutrients-4244275-supplementary- File S1.pdf]

Supplementary Material

File S1 Full electronic search strategy for PubMed, Scopus, Physiinfo, Web of science and EMBASE databases

PubMed

"Social Network"[All Fields] OR "Social Networking"[All Fields] AND (("obesity"[MeSH Terms] OR "obesity"[All Fields]) OR ("overweight"[MeSH Terms] OR "overweight"[All Fields])) AND (("adolescent"[MeSH Terms] OR "adolescent"[All Fields] OR "teenagers"[All Fields]) OR ("adolescent"[MeSH Terms] OR "adolescent"[All Fields] OR "adolescents"[All Fields])) AND ("2009/01/01"[PubDate] : "2022/12/31"[PubDate]).

Scopus

TITLE-ABS-KEY ("Social Network" OR "Social Networking") AND ("obesity" OR "overweight") AND ("adolescent" OR "teenagers") AND PUBYEAR > 2009 AND PUBYEAR < 2022

PsycInfo

“social networks” [Any Field] OR social networking [Any Field] AND adolescents [Any Field] OR teenagers [Any Field] AND obesity [Any Field] OR overweight AND APA Full-Text Only AND Peer-Reviewed Journals only AND Year: 2009 To 2022

Web of science

ALL=("Social Network"[All Fields] OR "Social Networking"[All Fields] AND (obesity OR overweight All Fields) AND adolescent OR teenagers)

Filtros 2009 a 2022

Embase

('social network'/exp OR 'social network' OR 'social networking'/exp OR 'social networking') AND ('obesity'/exp OR 'obesity' OR 'overweight'/exp OR 'overweight') AND ('adolescent'/exp OR 'adolescent' OR 'teenagers')

Filtros 2009 a 2022

The search was commenced in October 2022 and the last update was performed on December 2022.
