# Supplementary material for: The Influence of Social Networks on Adolescent Overweight and Obesity: A Narrative Review
Source: Nutrients. 2026 Jun 15;18(12):1930. doi: 10.3390/nu18121930 (PMC13305445; doi:10.3390/nu18121930)

Figure S1 PRISMA- Style flow diagram of study selection.The searches conducted in the electronic databases PubMed, Scopus, EMBASE, PsycINFO, and Web of Science yielded 10.884 records. After the removal of 76 duplicates, 10,808 articles remained, whose titles and abstracts were screened. Of these articles, 10.775 were excluded for not meeting the eligibility criteria. In the next stage, 33 articles were selected for full-text assessment. However, 26 of these articles were excluded: 7 because they did not link obesity to social network analysis, 8 because they were based on computational agent-based models, 4 because the age of the participants did not meet the inclusion criteria, and 7 because they assessed only BMI without linking it to dietary behaviors or physical activity. Thus, 7 articles were included in the systematic review. The screening process is detailed in Figure 2.

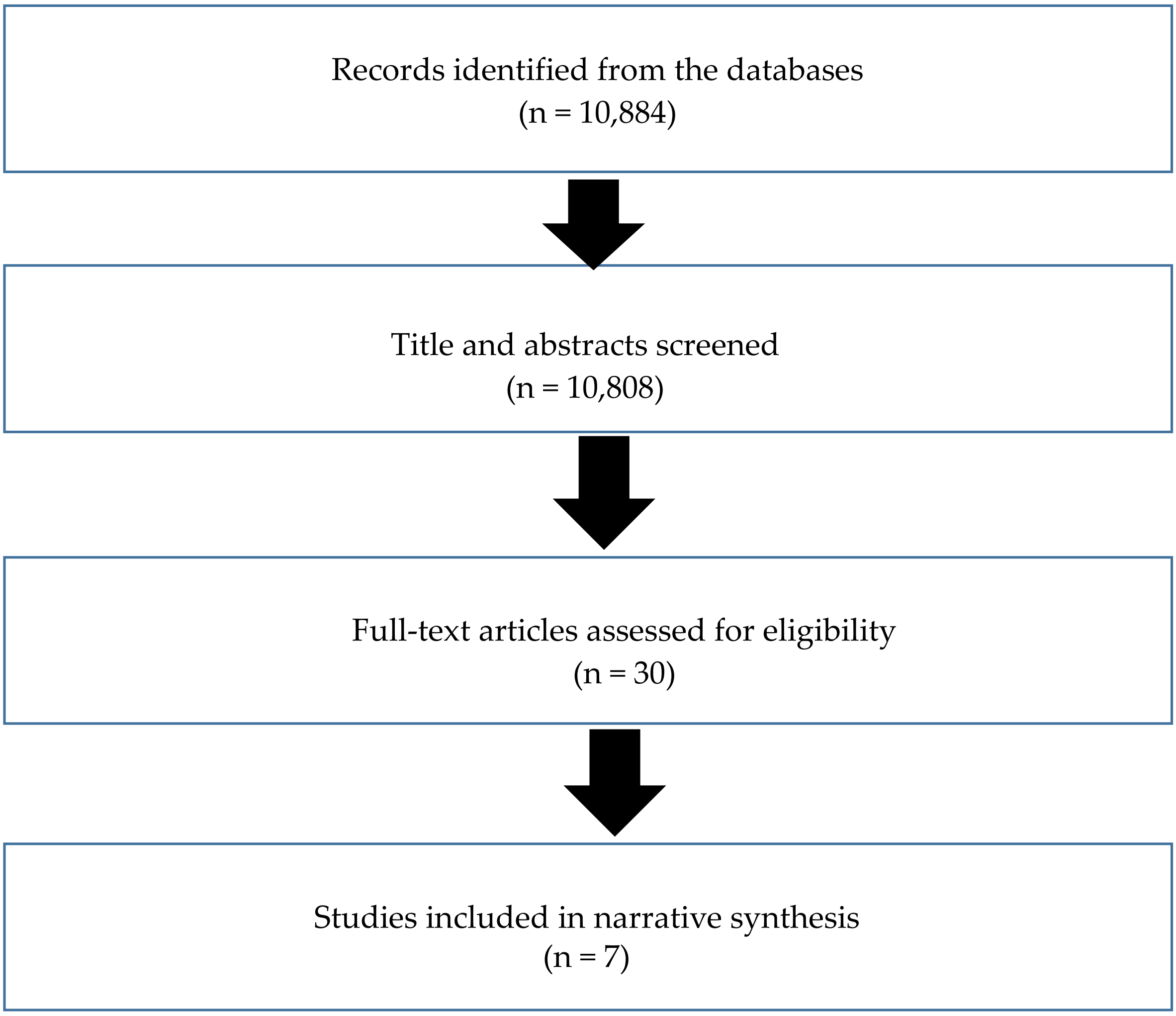

Supplement: Supplementary file 1 [file nutrients-18-01930-s001.zip › nutrients-4244275-supplementary-Figure S1.pdf]
